# Proprioception, Emotion and Social Responsiveness in Children with Developmental Disorders: An Exploratory Study in Autism Spectrum Disorder, Cerebral Palsy and Different Neurodevelopmental Situations

**DOI:** 10.3390/children11060719

**Published:** 2024-06-13

**Authors:** Inmaculada Riquelme, Samar M. Hatem, Álvaro Sabater-Gárriz, Elisabeth Martín-Jiménez, Pedro Montoya

**Affiliations:** 1 Department of Nursing and Physiotherapy, University of the Balearic Islands, 07122 Palma de Mallorca, Spain; 2 Research Institute on Health Sciences (IUNICS-IdISBa), University of the Balearic Islands, 07122 Palma de Mallorca, Spain; pedro.montoya@uib.es; 3 Health Research Institute of the Balearic Islands (IdISBa), 07010 Palma de Mallorca, Spain; 4 Faculty of Medicine, STIMULUS Research Group (reSearch and TeachIng neuroModULation Uz bruSsel), Vrije Universiteit Brussel, 1090 Brussels, Belgium; 5 Institute of Neuroscience, Université Catholique de Louvain, 1200 Brussels, Belgium; 6 Balearic ASPACE Foundation, 07141 Marratxí, Spain; elisamartin@aspaceib.org; 7 Center for Mathematics, Computation and Cognition, Federal University of ABC, São Bernardo do Campo 09606-045, Brazil

**Keywords:** proprioception, proprioceptive reactive behavior, emotion knowledge, emotion regulation, social responsiveness, neurotypical children, neurodevelopmental disorders, autism, cerebral palsy

## Abstract

Proprioception has long been linked with emotional dysregulation in neurotypical adults. Neuropediatric disorders such as autism spectrum disorder (ASD) and cerebral palsy (CP) are distinct entities and yet both present with deficits and challenges in sensory processing and the regulation of emotions. This study aimed to explore the relationship between proprioception and emotional–social performance in children and to compare proprioception and emotional–social performance in different underlying neurodevelopmental conditions. For this purpose, this cross-sectional study included 42 children with ASD, 34 children with CP and 50 typically developing peers. Proprioceptive acuity, proprioceptive reactive behavior as well as emotion regulation and social responsiveness were assessed. The results show a significant correlation between proprioceptive deficits and emotional difficulties in this pediatric sample, with distinct proprioceptive impairment patterns according to the underlying neurological disorder. Children with CP showed significant emotional knowledge deficits, while children with ASD predominantly showed challenges in social responsiveness. These data thus suggest a differentiated impact of proprioception on emotional–social performance in neurodevelopmental disorders and highlight proprioception as a potential therapeutic target for balancing emotion regulation in children with neurodevelopmental conditions.

## 1. Introduction

Proprioception is the information arising from the positional and movement afferents of the body. On the other hand, emotion perception difficulties are frequently associated with social responsiveness and everyday social functioning [1]. Recent emotion and embodiment theories consider proprioception an essential factor for the development of socioemotional processes. For example, proprioception has been related to the processing and regulation of somatic states which are implicated in the construction of emotion [2,3]. It is also thought that emotions are started and modulated through the perception of proprioceptive and interoceptive inputs [4]. Thus, position sense would be important in situations that include somatic as well as emotional responses. In this sense, brain structures where somatic, vestibular, visceral and exteroceptive sensory information converges, such as the parabrachial nucleus or the cerebellum, appear to be involved in the modulation of avoidance conditioning, anxiety and fear or the adaptation to environmental stimuli [5,6]. Moreover, facial muscles have been pointed to be essential for encoding and transmitting inputs to the neural emotional network, in a phenomenon known as emotional proprioception [7]. For instance, when frowning, the proprioceptive signal from facial muscles affects one’s emotional state, and makes individuals judge unpleasant stimuli as more negative [8]. Furthermore, the encoding of limb position and the creation of somatotopic maps have been related to the generation of behavior and social interactions [9]. In this sense, embodiment theories stress the role of sensory mechanisms in psychological and social processing, for example, the role of perception of body posture in bodily empathy [10,11]. In the opposite direction, the emotional state of an individual may influence the kinesthesic message in the adult neurotypical population, via the lengthening of the muscle spindles and the muscle afferent firing [12].

Neurodevelopmental disorders share common neurophysiological characteristics, such as their early origin, their impact on the developmental process of neural pathways and functioning and their consequences along the lifespan [13]. These shared traits have given place to theories considering neurodevelopmental disorders as a common condition with “cross-diagnostic boundaries (…) irrespective of syndrome-specific expectations” [13]. As an example of these cross-diagnostic characteristics, emotion dysregulation has been described as a cross-disorder trait in neurodevelopmental disorders, such as attention-deficit/hyperactivity disorder, autism spectrum disorder, oppositional defiant disorder/conduct disorder or anxiety or mood disorders [14]. Somatosensory impairments are also common features in most neurodevelopmental conditions, for example, Fragile X syndrome, autism spectrum disorder, attention-deficit/hyperactivity disorder or cerebral palsy [15,16,17,18]. In particular, proprioceptive deficits have been reported both in children with autism spectrum disorder (ASD) and in children with CP [19,20]. Nevertheless, the different neurodevelopmental conditions may have idiosyncratic expressions of these somatosensory deficits [14]. For example, proprioceptive impairments in children with CP have been associated with motor function impairment and spasticity [21,22,23], whereas a non-motor condition such as ASD has exhibited diminished proprioception in the form of altered speed-based proprioception [19,24,25]. The study of a priori such different conditions may give clues to the understanding of potential different physio-pathological mechanisms.

Although both proprioceptive and emotion regulation impairments have been reported in children with neurodevelopmental disorders [14,15,16,17,18], little research has investigated the interaction of these processes. Given the role of sensory information in developing appropriate emotional responses and social interaction [9], it is plausible that misleading proprioceptive information could generate “internal fake news” and provoke inappropriate or defective emotional adaptation to the environment [5]. The association between proprioception and emotion regulation has previously been reported in cerebellar or vestibular disorders in adults [5]. However, the relationship between proprioception and emotional and social performance in neurodevelopmental disorders has been investigated scarcely. One study has described an association between proprioceptive function and communicative autistic traits in a sample of neurotypical adult subjects [26]. In children with ASD, it has been shown that social stimuli (viewing images of human faces vs. images of objects) affected postural control [27]. One should keep in mind that posture and balance rely on more than solely proprioceptive input; they depend on complex interactions between different sensory inputs (e.g., proprioceptive, visual and vestibular inputs) and motor performance [28]. With the purpose to add novel evidence on the interaction between proprioception and socioemotional function in different pediatric populations, this study aims to explore the relationship between proprioception and emotional–social performance in children and to compare proprioception and emotional–social performance impairments in different underlying neurodevelopmental conditions (autism spectrum disorder, cerebral palsy). We hypothesize that children with neurodevelopmental conditions will have impaired proprioception compared to typically developing peers and that their proprioceptive deficits will be linked with emotional and social impairments. Increasing the knowledge on how these constructs are associated may lay the basis for future research and clinical protocols including proprioception into the aspects that contribute to socioemotional difficulties.

## 2. Materials and Methods

### 2.1. Participants

Participants diagnosed with autism spectrum disorder (ASD) according to DMS criteria (DSM 5) or with a diagnosis of cerebral palsy (CP) (diagnosis reported by a neurologist in the medical history) were recruited from early care centers and patient associations in Majorca (Spain). These two developmental conditions were chosen as they present completely different symptoms distinctly related to the variables of the study: social impairments in the case of children with ASD, and a motor problem in the case of children with CP that may directly affect proprioception. Inclusion criteria were as follows: age between 4 and 18 years. For participants with ASD, a severity level 1 or 2 of the DSM-5 (verbal expression at least of simple sentences, requiring low-medium support) was required. For participants with CP, children were required to be able to understand and perform the tasks of the study (as assessed by the psychologist of their care center). Exclusion criteria were as follows: severe cognitive impairment, not allowing to understand the tasks; severe motor, sensory or communication problems, not allowing to execute the tasks. Sex- and age-matched typically developing peers (TDPs), without a diagnosis of neurodevelopmental disorders, were recruited from ordinary schools, through advertising of the study among parents and teachers; also, the care centers and patient associations involved in the recruitment of children with ASD or CP made a call for typically developing volunteers. The sample size was calculated taking proprioceptive acuity measured with the Nottingham Sensory Assessment as primary outcome. Previous studies in children with ASD showed a difference between groups of 0.5 units, with a common standard deviation of 0.43 [18]. Applying an alpha risk of 0.05, a confidence level of 95%, and a loss rate of 10%, the sample size would be of 27 children per group. We planned to recruit at least 30 children per group in order to ensure we could detect differences among the other variables.

Recruitment was performed following the standardized procedure used in other studies of our lab [19,20]. Children meeting inclusion and exclusion criteria were selected by professionals from 9 entities (4 patient associations and 5 early care centers) in the case of children with CP or ASD. A letter informing about the study was sent to potential participants through the centers’ social media. Families interested in participating provided their contact details through an online platform. Families of typically developing children directly contacted the research team by providing their details through this platform. Subsequently, a member of the research team contacted them by phone or email, provided information about the study and answered their doubts. In case families were interested in participating, they received an e-mail with a written consent form and the study questionnaires, with the instructions to complete them and a date for the child’s assessment. In this session, parents brought along the signed written informed consent and the questionnaires, which were revised to solve doubts and avoid blanks. The examiner explained the objectives and tasks of the study to the children in a child-friendly way. All children gave their oral approval to participate. Children completed the Emotion Matching Task (EMT), and their proprioceptive acuity was measured by an experienced researcher. Augmentative communication devices and information from caregivers were used as needed, to facilitate data collection in participants with communication difficulties. Parents and participants gave their consent to use clinical data from the medical health reports to characterize the children (level of severity, language function, motor function, existence of chronic pain defined as persistent pain lasting more than 3 months). Cognitive function was extracted from the psychological health report. It was classified into mild, moderate and severe impairment based in the criteria of DSM-V, reflecting the child’s conceptual, social and practical ability.

The Ethics Committee on Research from the University of the Balearic Islands (ref. 127CER19) approved the study. Patient associations and professionals of the participant care centers for autism spectrum disorder and cerebral palsy were involved in developing the potential adaptations of research questions and measurements, as well as in advertising the study among families and disseminating results. No compensation was given for participation.

### 2.2. Measures

#### 2.2.1. Proprioception Assessment

Proprioceptive acuity was assessed using two tasks: the proprioceptive task of the Nottingham Sensory Assessment [29] and the Joint Position Error [30]. For facilitating adequate active movement in children with motor impairments, both assessments were performed on the dominant wrist (or less affected side in the case of children with CP). Participants were seated with their elbows flexed at 90° and forearms in pronation. They were blindfolded during proprioceptive tasks. Joint movements were performed at established angles of 5, 10, 15, 30, 45 and 60° from the neutral position and in a randomized order. These different angles of joint range-of-motion were chosen to avoid a bias due to joint angle, direction and range of motion [31].

The proprioceptive task of the Nottingham Sensory Assessment (NSA) assesses proprioception by reproducing passive joint movements performed by the experimenter. The original proprioceptive task may require that the participant express or mirror the change of movement with the contralateral limb. For the present study, the task was adapted to accommodate participants with motor impairment and the participant was asked to verbally identify the direction of joint movement and the joint’s end position. This adaptation has been used previously in adults with cerebral palsy [32] and in children with ASD [19,33]. The wrist was passively positioned in one of four wrist positions (palmar flexion, dorsal flexion, radial deviation and ulnar deviation) and children were asked to describe the direction of the movement and the end position of the wrist. Proprioception was scored according to the following criteria: 2 = normal, ability to describe final joint position within 10° range of error; 1 = partially impaired, ability to appreciate joint movement but failure to detect movement direction; 0 = impaired, no appreciation of joint movement. The final score was the sum of the four scores of the different wrist movements. A higher score on the test indicated better proprioception (range 0–16). This task has demonstrated good intra- and inter-rater reliability for the upper limb (weighted κ = 0.62–1.00 and κ = 0.48–1.00, respectively) [29].

The wrist joint position error was measured using an active joint position reproduction test. The joint position error quantifies the ability to reproduce a joint’s position following a passive movement performed by the examiner [30]. For this test, the hand was placed over a plane with marked radial angles. The examiner performed a passive wrist movement at a constant velocity in one of the previously mentioned directions. The wrist remained in that position for 10 s so that the children could remember it, and, consecutively, the wrist was passively moved to the starting position. After staying at the starting position for 5 s, the participant was asked to move the wrist to match the target position actively. The absolute difference between the perceived angle indicated by the children and the previous position by the examiner was defined as the absolute angular error expressed in degrees (°). The measurement has shown a test–retest reliability of 0.59 [34]. This procedure was repeated for each of the four directions (palmar flexion, dorsal flexion, radial deviation and ulnar deviation). The sum of the errors in each of the four wrist positions was regarded as the joint position error, with higher values indicating worse proprioception. This method for assessing proprioception has previously been used in individuals with cerebral palsy [35].

The proprioceptive reactive behavior, defined as emotional behavior produced in response to situations with high proprioceptive/vestibular inputs, was assessed with the Subscale Movement of The Short Sensory Profile. The Short Sensory Profile consists of a 38-item questionnaire answered by parents, to evaluate sensory processing dysfunction in children and adolescents [36,37]. It provides information on 7 domains (taste/smell, tactile, movement, auditory filtering, low energy, visual and auditory sensitivity and under-responsiveness/sensation seeking). Each item can be rated on a 5-point Likert scale (1 = always, 5 = never), with lower scores expressing more sensory-reactive behaviors. Items of the Subscale Movement explore reactivity to high-proprioceptive/vestibular situations, such as anxiety when feet are separated from the floor, fear of falling or dislike of activities where the head is upside-down. This tool has good psychometric properties (Cronbach’s α = 0.70–0.90) [38] and has been used previously in children with ASD and children with CP [39,40].

#### 2.2.2. Emotion and Social Assessment

Emotion Matching Task (EMT). This test measures the emotion knowledge of happiness, sadness, anger and fear/surprise in children through 4 different tasks, evaluated through the observation of photographs of children with different affective facial expressions: EMT1 (expression matching), EMT2 (emotion situation knowledge), EMT3 (expressive emotion knowledge) and EMT4 (receptive emotion knowledge). The score of each task is calculated by adding up the number of correct answers (maximum score = 12). Both the original version and the Spanish version used in this study present good psychometric properties for each of the tasks (α = 0.81–0.88 and 0.82–0.94, respectively) [41,42]. The EMT has been previously used for studying emotion knowledge in children with ASD and children with CP [39,43].

Emotion Regulation Checklist. This caregiver questionnaire is composed of 24 items, answered by a 4-point Likert scale (never/almost never/almost always/always). The questionnaire provides information on two subscales: emotional lability/negativity and emotional regulation. High scores in the subscale emotional lability/negativity indicate emotional dysregulation, whereas high scores in the subscale emotional regulation and in the total score indicate a good capacity for regulating emotions. This tool has high psychometric properties (emotional regulation α = 0.83, emotional lability α = 0.96) [44] and has been used previously for studying emotion regulation in children with ASD and children with CP [39,43].

The Social Responsiveness Scale (parents’ version) [45] is a 65-item questionnaire which assesses social communication and restricted repetitive behaviors and interests. It is composed of 65 items, rated on a 4-point Likert scale. The total score is calculated by the sum of the scores and may yield a maximum total score of 195. Five domains of social responsiveness were additionally calculated: social motivation, social awareness, social cognition, social communication and the presence of mannerisms. The scores of the total scale and each domain were separately used in this study, with higher values indicating greater social impairments. This questionnaire has good psychometric properties (α = 0.80) [46] and scores that are not related to the child’s cognitive capacity or age [47].

#### 2.2.3. Statistical Analysis

Descriptive statistics were computed. Kolmogorov–Smirnov tests showed an abnormal distribution for all experimental variables (*p* < 0.001); therefore, non-parametric tests were used for further statistical analysis. Spearman correlations and Mann–Whitney tests were used to assess the influence of age, sex and chronic pain on proprioception variables. Spearman correlations were computed to assess the relationship between proprioceptive and emotional–social variables in the entire study population. Correlations within groups were not performed due to the smaller sample size. Non-parametric one-way ANOVA tests on ranks (Kruskal–Wallis) were used for assessing the interaction between the factor GROUP (ASD vs. CP vs. TPD) and each variable. For further analyzing the effects of age and sex on proprioception, ANCOVAs including age or sex as covariates were performed for the proprioception variables. Pair-wise comparisons were computed with post hoc Bonferroni. Significance level was set at *p* < 0.05 for all statistical tests.

## 3. Results

### 3.1. Participants

Forty-two children with autism spectrum disorder [12 girls; Mean age = 11.45 yrs (SD = 4.24)], 34 children with cerebral palsy [10 girls, Mean age = 14.25 yrs (SD = 3.83)] and 50 typically developing peers [24 girls; Mean age = 9.76 yrs (SD = 3.19)] participated in the study. The clinical characteristics of all participants are displayed in Table 1. No correlations were found between age and proprioception variables, except for proprioceptive reactive behavior (Short Sensory Profile Scale) (rho = −0.236, *p* = 0.010) where younger age was linked with less movement-reactive behaviors. The presence of chronic pain had a significant influence on proprioception (scores in the proprioceptive task of the Nottingham Sensory Assessment, Mann–Whitney U = 469.50, *p* = 0.006); children with chronic pain had poorer proprioception than children without pain. Proprioception measurements were not different between girls and boys (all *p* > 0.115).

### 3.2. Correlations between Proprioceptive and Socio-Emotional Performance in the Entire Study Population

**Figure 1:**
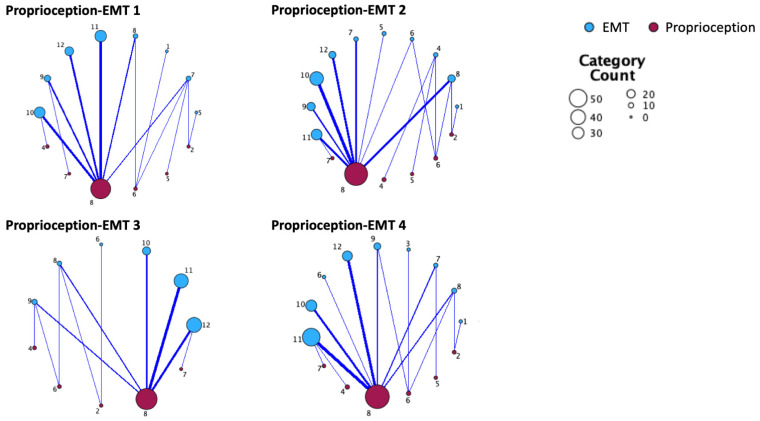
Relationship maps between proprioception acuity and emotion knowledge. EMT: emotion matching task (EMT 1: expression matching, EMT 2: emotion situation knowledge, EMT 3: expressive emotion knowledge, EMT 4: receptive emotion knowledge). Proprioceptive acuity is displayed according to the scores in the proprioceptive task of the Nottingham Sensory Assessment. Thicker lines represent stronger relationships, bigger circle size represents higher number of children in the category.

Scores of the proprioceptive task of the Nottingham Sensory Assessment were significantly correlated with each of the EMT tasks (all rho > 0.45, all *p* < 0.001). Scores of the Joint Position Error task were significantly correlated with EMT1, EMT3 and EMT4 (all rho > −0.29, all *p* < 0.046). Scores of proprioceptive reactive behavior (Short Sensory Profile Scale) were significantly correlated with EMT3 (rho = 0.24, *p* = 0.023). Taken together, these results indicate that poorer proprioceptive performance is related to lower emotion knowledge (Figure 1).

Scores of proprioceptive reactive behavior were also significantly correlated with emotion lability (Emotion Regulation Checklist) (rho = −0.22, *p* = 0.046). This suggests that increased reactive behaviors due to movement are linked with a higher emotion lability/negativity.

No correlations were observed between proprioceptive measurements and social responsiveness variables.

### 3.3. Comparison of Proprioception, Emotion Regulation and Social Responsiveness between Groups of Children with Autism Spectrum Disorder, with Cerebral Palsy and Typically Developing Peers

Table 2 shows the proprioception variables for each group. The score of the proprioceptive task of the Nottingham Sensory Assessment was significantly different between groups (Kruskal–Wallis F(2,59) = 17.19, *p* < 0.001). Post hoc pairwise comparisons indicated that children with CP had poorer proprioceptive acuity in comparison with TDP and with children with ASD (both *p* < 0.001), whereas no significant differences were found between children with ASD and TDP (*p* = 0.720). No significant differences between groups were observed for the Joint Position Error (F(2,56) = 1.48, *p* = 0.478). The score of proprioceptive reactive behavior (Short Sensory Profile Scale) was significantly different between groups (F(2,118) = 10.00, *p* = 0.007). Post hoc pairwise comparisons indicated that children with CP had lower scores than children with ASD (*p* = 0.002), whereas no significant differences were found between children with CP and TDP or between children with ASD and TDP (both *p* < 0.054). When introducing age as a covariate, the significant differences in proprioceptive reactive behavior disappeared (*p* = 0.061), while previous statistical effects on the proprioceptive task of the Nottingham Sensory Assessment and Joint Position Error were maintained (*p* = 0.003 and *p* = 0.804, respectively). When introducing age as a covariate, the significant differences in proprioceptive reactive behavior disappeared (*p* = 0.061), while previous statistical effects on the proprioceptive task of the Nottingham Sensory Assessment and Joint Position Error were maintained (*p* = 0.003 and *p* = 0.804, respectively). No differences in significances were found when sex was introduced as a covariate.

Table 3 shows the outcomes of emotion and social variables for each group. The score of the Emotion Matching Task was significantly different between groups in each of the four tasks (all F > 12.99, all *p* < 0.01). Post hoc pair-wise comparisons indicated that children with CP had lower hits (lower emotion knowledge) than their TDPs and than children with ASD in all the tasks (all *p* < 0.017), whereas no significant differences were found between children with ASD and TDPs. No significant differences between groups were observed for the Emotion Regulation Checklist, neither in the regulation nor the lability domain (both *p* > 0.245).

The total score of the Social Responsiveness Scale was significantly different between groups (F(2,58) = 19.61, *p* < 0.001). Post hoc pair-wise comparisons indicated that children with ASD had greater social impairments than children with CP and than TDPs for the total score (both *p* < 0.010). Regarding the five domains of the Social Responsiveness Scale, children with ASD showed greater impairment than children with CP and TDPs in the domains of social communication and mannerism (all *p* < 0.01). Children with ASD showed greater impairment in the domains of social cognition and social motivation compared to TDPs (both *p* < 0.002), but not compared with children with CP (*p* > 0.079). No significant differences between groups were found in the domain of social awareness. Pair-wise comparisons showed no differences between children with CP and TDPs in any of the domains, except mannerism (*p* = 0.016).

## 4. Discussion

The present study aimed to explore the relationship between proprioception and emotional–social performance in children and to compare proprioception and emotional–social performance impairments in different neurodevelopmental situations (ASD, CP, TDPs). In children, proprioception deficits were linked with impairments in emotion knowledge and emotion lability/negativity, but not with social responsiveness. The presence of chronic pain and age, but not sex, had a significant influence on proprioception. The findings further revealed that children with CP presented with abnormal proprioception compared to TDPs and children with ASD. Proprioceptive reactive behaviors, but not proprioceptive acuity, were mediated by age, with only younger children showing less movement-reactive behaviors. Children with ASD had a similar proprioceptive performance as TDPs. Regarding emotional–social performance, children with CP were significantly different from children with ASD: children with CP rather showed impairments in emotion knowledge, while children with ASD had impairments in social responsiveness.

Our study adds novel evidence that highlights proprioception as a paramount element linked with emotional function in children. Sensory processing features have previously been pointed out as key elements for emotion regulation and behavior [39] and for the development of social skills during childhood and adolescence in children with ASD [48,49,50]. In particular, the perception of bodily signals has been pointed out as a facilitator of the use of strategies to regulate emotions and the ductile selection of the adequate behavior according to social context in neurotypical individuals [51]. The perception of body posture, kinesthesia and limb position are regarded as important factors for the regulation of psychological states in neurotypical populations [9,10,11]. It has also been proposed that inadequate proprioceptive information in neurodevelopmental disorders may generate a distorted regulation of somatic states implicated in the construction of emotional processes, provoking deficiencies in the emotional adaptation to the environment [3,5]. Abnormal somatotopic representation and altered attention to interoceptive inputs affect socio-affective functions, such as empathy [52,53], and are linked to emotion-regulation deficits and social anxiety in populations with ASD [54,55]. This evidence has given rise to some therapies based on proprioception and movement perception to treat emotional dysregulation states, such as depressive disorders, in adults. For example, therapies that manipulate facial muscles or dance/movement (psycho)therapy use proprioception therapeutically to help regulate emotions in the neurotypical population [56,57]. At present, many therapists use proprioceptive rehabilitation for improving emotional regulation in children. Clinics all over the world and commercial websites advertising these therapies abound. Rehabilitation includes body awareness, self-regulation, posture, weighted blankets, etc. They build upon the available literature in the adult population. However, few scientific arguments are available in the pediatric population to back up on proposed therapies and provide a direct link between proprioception and emotion regulation [26,27]. The present study contributes to the development of a framework considering emotional impairments in neurodevelopmental disorders on a broader scale, including proprioception into the aspects of body awareness contributing to emotional difficulties. This framework implies a broad assessment of emotional impairments, from basic constructs such as emotion knowledge to more complex emotional abilities, together with the assessment of bodily perception, including proprioception, exteroception, interoception and body representation.

Although one of our hypotheses was that proprioception would be related with social responsiveness, our data did not confirm this assumption. This was a surprise, as the encoding of limb position, the perception of body postures and the creation of somatotopic maps have been associated with social understanding and the generation of social interactions [9,10]. Moreover, proprioceptive deficits have been related to autistic-like social communication [26]. The framework of Hoffman’s Social Deafferentation Hypothesis, applied in other pathologies, such as schizophrenia, proposes that proprioception contributes to the sense of body boundary, which contains the self and is influenced by social interactions; this implies that the anomalous bodily experiences may lead to a withdrawal of social environment and may lead to a maladaptive plasticity of the social brain network [58]. In this sense, some brain structures implied in the processing of proprioceptive inputs are also connected with brain regions involved in social cognition. Studies with animal models have shown that cerebellar disturbances reduced the preferences for social interactions [59]. Brain regions such as the dorsal medial–frontal cortex and the anterior cingulate monitor proprioceptive information concerning self-action and integrate it with exteroceptive inputs about the behavior of others; an anormal function of these structures may contribute to the atypical development of social cognition [60]. It is likely that our assessment did not have the capacity for unveiling these complex interaction processes, affecting more complex concepts such as the construction of the body self and the representation of others. More research is warranted to deepen the understanding of the mechanisms relating proprioception and social behavior in children development.

The present study explored how similar (or different) the impairment patterns of proprioception, emotion knowledge/regulation and social responsiveness were across distinct neurodevelopmental disorders such as ASD and CP. Theoretically, in neurodevelopmental disorders, cross-diagnostic boundaries and characteristics could be found irrespective of syndrome-specific characteristics [13]. In the present study, population, children with CP showed significant proprioceptive and emotional knowledge deficits, while children with ASD predominantly showed impairments in social responsiveness. Proprioceptive deficits have previously been reported in different neurodevelopmental conditions such as children with developmental coordination disorder [61], dystonia [62], dyslexia [24], CP [21] or ASD [63]. Extensive research has reported proprioceptive impairments in children with CP [21,22,23,64,65], impacting activity, participation and functional performance [22,23]. Proprioceptive deficits in children with CP have been associated with motor function impairment and spasticity [25,64,66] and have been attributed to abnormalities of spino- and thalamo-cortical neural tracts, abnormal cortical activity and lower muscle H-reflex excitability [35,66,67]. On the other hand, previous research in children with ASD has reported diminished proprioceptive performance, in the form of poor perception of joint position and altered speed-based proprioception [19,63,68]. These clinical findings have been corroborated by studies showing alterations in neural networks processing proprioception, such as the inferior parietal lobe, in children with ASD [69]. The present findings confirmed poorer proprioception acuity and proprioceptive reactive behaviors in children with CP, which can be explained by the influence of spasticity on muscle lengthening (which are characteristic of the CP condition), as well as the abnormalities in neural tracts and afferent cortical perception [35,64]. Surprisingly, our results do not show abnormal proprioception in children with ASD, when compared to TDPs. Complex proprioceptive abnormalities, such as internal sensorimotor representations biasing proprioceptive over visual feedback, or the integration of proprioception with other sensory information, have been reported in children with ASD [70,71]. This may be due to the fact that the proprioceptive deficits attributed to children with ASD by previous studies could affect more complex neural integration mechanisms, not assessed with our measures. Thus, although proprioceptive impairment may be a cross-disorder trait, its specific expression in function of the underlying disorder warrants specific and appropriate assessments. The influence of pain and age in the modulation of proprioception is noteworthy. In our study, children with chronic pain had poorer proprioception than children without pain and age-modulated differences in proprioceptive reactive behaviors. These results are in concordance with previous studies reporting impaired proprioception in chronic pain conditions in adults with musculoskeletal injuries [72,73] and a physiological neurodevelopmental adaptation to the responsivity to somatosensory stimuli during childhood [74]. As these effects seem to also affect children with neurodevelopmental disorders, they must be taken into account in the design of assessment protocols and interventions.

Emotion/social dysregulation was found both in children with CP and with ASD, nonetheless with different clinical expressions. Children with CP showed impairments in more basic aspects of the psychosocial process, such as emotion knowledge. Deficits in emotion knowledge have previously been reported in children with CP [43] as well as in other neurodevelopmental conditions, such as attention-deficit/hyperactivity disorders [75]. Emotion knowledge plays an essential role in social functioning and psychosocial adaptation, making it possible to interpret the emotional data from social interactions [76,77]. In contrast, the present sample of children with ASD did not show impairment in recognizing emotions in others’ faces, but rather in social responsiveness. Less basic parameters of emotion, such as empathy or the theory of mind, have been shown to be impaired in populations with ASD [48,78]. This may limit their social competence. Though the specific elements of abnormal emotion processing differ between children with ASD and CP, they still have the common consequence of impacting the emotional regulation process in both conditions.

Limitations: the assessments used to evaluate proprioceptive performance may have lacked sensitivity for more complex aspects of proprioception and proprioceptive processing, such as, for example, proprioception integration or its role in interoception or social behavior. Also, some specific aspects of psychosocial function, such as empathy, could have helped clarify the relationship between body awareness, perception of others and social function. The low number of girls in the groups, although in accordance with the prevalence of ASD and CP conditions, may have masked the statistical effects of sex on proprioception. These variables could provide interesting information and should be taken into account in future studies.

In summary, proprioceptive impairments are linked with emotional functioning both in neurotypical children and in children with neurodevelopmental disorders though specific disorder-related differences exist. The innovative contribution of the present findings underscores the importance of proprioception for emotional function. Our results not only provide a framework for future studies relating both areas, but also strongly support the recommendation of assessing proprioception deficits as a part of the clinical characterization of emotional impairment in children.

## Figures and Tables

**Table 1:** Clinical characteristics of children with ASD and children with cerebral palsy. ASD: Autism spectrum disorders, CP: Cerebral palsy, TDP: Typically developing peers.

Variable (n)	Children with ASD (n = 42)	Children with CP (n = 34)	TDP (n = 50)
**Chronic pain**	7	17	3
**Cognitive function**			
Normal cognition	34	20	50
Mild impairment	4	8	0
Moderate impairment	4	6	0
Severe impairment	0	0	0
**Language function**			
Fluid language	27	22	50
Some sentences or echolalia	4	0	0
Some words	5	4	0
Non-verbal	6	8	0
**Type of ASD**		-	-
Level 1	25		
Level 2	17		
**Type of CP**			
Bilateral spastic	-	24	-
Diskinetic	-	7	-
Ataxic	-	3	-
**Gross motor function classification system**			
Level I	42	2	50
Level II	0	6	0
Level III	0	10	0
Level IV	0	6	0
Level V	0	10	0
**Manual ability function classification system**			
Level I	42	6	50
Level II	0	6	0
Level III	0	6	0
Level IV	0	6	0
Level V	0	10	0

**Table 2:** Proprioception in children with ASD, children with cerebral palsy and their typically developing peers. Non-parametric one-way ANOVA tests on ranks (Kruskal–Wallis), with post hoc pair-wise comparisons (*p* value), for the factor GROUP (ASD vs. CP vs. TPD). ASD: Autism spectrum disorders, CP: Cerebral palsy, TDPs: Typically developing peers. * *p* < 0.05.

	Children with ASD (n = 42)	Children with CP (n = 34)	TDPs (n = 50)	Statistical Effects
	Mean (SD)	Mean (SD)	Mean (SD)	*p* Value/Effect Size
Proprioception task (Nottingham Sensory Assessment)	7.94 (0.24)	6.14 (2.48) *	8.00 (0.00)	ASD-TDP: *p* = 0.720/d = 0.34 CP-TDP: *p* < 0.001/d = 1.03 ASD-CP: *p* < 0.001/d = 0.98
Joint Position Error (° of error)	23.39 (25.40)	27.32 (29.91)	17.95 (15.82)	ASD-TDP: *p* = 0.684/d = 0.26 CP-TDP: *p* < 0.231/d = 0.39 ASD-CP: *p* < 0.439/d = 0.14
Proprioceptive reactive behavior (Short Sensory Profile)	12.98 (2.91)	10.73 (3.29) *	12.08 (3.46)	ASD-TDP: *p* = 0.124/d = 0.27 CP-TDP: *p* = 0.054/d = 0.40 ASD-CP: *p* = 0.002/d = 0.73

**Table 3:** Emotion knowledge, emotion regulation and social responsiveness in children with ASD, children with cerebral palsy and their typically developing peers. Non-parametric one-way ANOVA tests on ranks (Kruskal–Wallis), with post hoc pair-wise comparisons (*p* value) for the factor GROUP (ASD vs. CP vs. TPD) and Cohen d for effect sizes. In EMT, higher scores reflect better emotion knowledge; in responsiveness, higher scores reflect higher social impairment. ASD: Autism spectrum disorders, CP: Cerebral palsy, TDP: Typically developing peers. Asterisks (*) mark differences with TDP. * *p* < 0.05, ** *p* < 0.01, *** *p* < 0.001.

	Children with ASD	Children with CP	TDP	Statistical Effects
	Mean (SD)	Mean (SD)	Mean (SD)	*p* Value/Effect Size
Emotion knowledge task				
EMT1 (emotion matching)	10.27 (1.66)	8.00 (2.73) ***	10.34 (1.33)	ASD–TDPs: *p* = 0.872/d = 0.53 CP–TDPs: *p* < 0.001/d = 1.29 ASD–CP: *p* < 0.001/d = 1.04
EMT2 (situational knowledge)	9.39 (2.19)	6.79 (2.66) ***	10.12 (1.29)	ASD–TDPs: *p* = 0.229/d = 0.45 CP–TDPs: *p* < 0.001/d = 1.89 ASD–CP: *p* < 0.001/d = 1.08
EMT3 (expressive knowledge)	10.77 (1.03)	9.31 (1.78) **	11.27 (0.87)	ASD–TDPs: *p* = 0.054/d = 0.54 CP–TDPs: *p* < 0.001/d = 1.67 ASD–CP: *p* = 017/d = 1.07
EMT4 (receptive knowledge)	10.44 (1.45)	8.42 (2.89) ***	10.70 (1.05)	ASD–TDPs: *p* = 0.588/d = 0.21 CP–TDPs: *p* < 0.001/d = 1.28 ASD–CP: *p* = 0.006/d = 0.92
Emotion regulation checklist				
Emotion regulation	3.13 (0.66)	3.27 (0.52)	3.20 (0.74)	ASD–TDPs: *p* = 0.488/d = 0.13 CP–TDPs: *p* = 0.968/d = 0.10 ASD–CP: *p* = 0.609/d = 0.22
Emotion lability/negativity	1.72 (0.66)	2.05 (0.69)	1.80 (0.59)	ASD–TDPs: *p* = 0.395/d = 0.10 CP–TDPs: *p* = 0.320/d = 0.40 ASD–CP: *p* = 0.096/d = 0.49
Social responsiveness scale				
Social motivation	17.22 (6.00) ***	13.67 (5.58)	10.95 (5.40)	ASD–TDPs: *p* < 0.001/d = 1.10 CP–TDPs: *p* = 0.055/d = 0.50 ASD–CP: *p* = 0.079/d = 0.62
Social awareness	12.72 (3.10)	11.09 (3.89)	11.53 (2.65)	ASD–TDPs: *p* = 0.355/d = 0.42 CP–TDPs: *p* = 0.798/d = 0.14 ASD–CP: *p* = 0.588/d = 0.48
Social cognition	19.50 (5.13) **	17.10 (5.17)	14.26 (3.11)	ASD–TDPs: *p* = 0.002/d = 1.24 CP–TDPs: *p* = 0.110/d = 0.66 ASD–CP: *p* = 0.125/d = 0.47
Social communication	35.22 (10.03) **	24.14 (10.14)	21.21 (6.42)	ASD–TDPs: *p* < 0.001/d = 1.42 CP–TDPs: *p* = 0.415/d = 0.34 ASD–CP: *p* = 0.001/d = 1.10
Mannerism	23.27 (6.92) ***	13.67 (5.58) *	6.32 (5.76)	ASD–TDPs: *p* = 0.016/d = 2.60 CP–TDPs: *p* < 0.001/d = 1.04 ASD–CP: *p* = 0.006/d = 1.48
Total score	107.67 (27.85) ***	80.05 (26.96)	64.26 (20.22)	ASD–TDPs: *p* < 0.001/d = 1.79 CP–TDPs: *p* = 0.055/d = 0.66 ASD–CP: *p* < 0.009/d = 1.01

## Data Availability

The original contributions presented in the study are included in the article.
